# Network Analysis of Sensory Phenomena and Obsessive‐Compulsive Symptoms in OCD: A Multicenter Study

**DOI:** 10.1155/da/1367751

**Published:** 2026-08-26

**Authors:** Yue Sun, Xuedi Zhang, Wenxin Tang, Qianqian Li, Jun Yan, Si Mi, Zhanjiang Li, Bin Li, Guiyun Xu, Congwen Yang, Maorong Hu, Zhenqing Zhang, Yanbin Jia, Zhen Tang, Xiaoping Wang, Jun Ma, Changhong Wang, Wei Liu, Na Liu

**Affiliations:** ^1^ Nanjing Brain Hospital Affiliated to Nanjing Medical University, Nanjing, Jiangsu, China, njmu.edu.cn; ^2^ Affiliated Mental Health Center and Hangzhou Seventh People’s Hospital, Zhejiang University School of Medicine, Hangzhou, Zhejiang, China, zju.edu.cn; ^3^ NHC Key Laboratory of Mental Health (Peking University Sixth Hopital), National Clinical Research Center for Mental Disorders (Peking University Sixth Hospital), Peking University Sixth Hospital, Peking University Institute of Mental Health, Beijing, China, pku.edu.cn; ^4^ Center of Clinical Psychology, Beijing Anding Hospital, Capital Medical University, Beijing, China, ccmu.edu.cn; ^5^ Beijing Key Laboratory of Mental Disorders, National Clinical Research Center for Mental Disorders and National Center for Mental Disorders, Beijing, China; ^6^ Mental Health Center, West China Hospital, Sichuan University, Chengdu 610041, China, scu.edu.cn; ^7^ The Brain Hospital Affiliated to Guangzhou Medical University, Guangzhou, China; ^8^ School of Psychology, Guizhou Normal University, Guiyang, China, gznu.edu.cn; ^9^ The 1st Affiliated Hospital, Jiangxi Medical College, Nanchang University, Nanchang, Jiangxi, China, ncu.edu.cn; ^10^ Xiamen Xianyue Hospital, Xianyue Hospital Affiliated With Xiamen Medical College, Fujian Psychiatric Center, Fujian Clinical Research Center for Mental Disorders, Xiamen, Fujian, China; ^11^ Department of Psychiatry, The First Affiliated Hospital of Jinan University, Guangzhou, China, jd120.com; ^12^ The Affiliated Guangji Hospital of Soochow University, Suzhou, Jiangsu, 215137, China; ^13^ Department of Psychiatry, National Clinical Research Center for Mental Disorders, The Second Xiangya Hospital of Central South University, Changsha 410011, Hunan, China, csu.edu.cn; ^14^ Pediatric and Adolescent Health Ward, Wuhan Mental Health Center, Wuhan, China; ^15^ Henan Collaborative Innovation Center of Prevention and Treatment of Mental Disorder, The Second Affiliated Hospital of Xinxiang Medical University, Xinxiang, China, hnjsby.cn; ^16^ Department of Psychiatry, The First Affliated Hospital of Harbin Medical University, Harbin, China, hrbmu.edu.cn; ^17^ Department of Medical Psychology, The Affiliated Brain Hospital of Nanjing Medical University, Nanjing, Jiangsu, China, c-nbh.com

**Keywords:** multicenter study, network analysis, obsessive-compulsive disorder, sensory phenomena, subtypes

## Abstract

**Background:**

Sensory phenomena (SP) affect 60%–80% of obsessive‐compulsive (OC) disorder (OCD) patients, demonstrate dimension‐specific symptom associations, yet their mechanistic role in OCD’s network architecture remains unexamined.

**Methods:**

In this cross‐sectional study, 1350 clinician‐diagnosed OCD patients from the China OCD Cohort (COCC) underwent standardized assessments using the University of São Paulo SP Scale (USP‐SPS), Yale‐Brown OC Scale (Y‐BOCS), OC Inventory‐Revised (OCI‐R), and Beck Depression Inventory‐II/Beck Anxiety Inventory (BDI‐II/BAI). Network analysis was utilized to identify core/bridge symptoms and test network differences across early/late‐onset and SP‐severity subgroups.

**Results:**

SP‐positive OCD patients (*n* = 850) demonstrated earlier disease onset (*p*  < 0.001), more severe compulsions (*p* = 0.010), and greater depression than SP‐negative patients (*n* = 500). Network analysis in the SP‐positive cohort identified SP symptoms as central network drivers, though with considerable heterogeneity across subtypes: muscle‐joint/bone sensation (strength = 3.41) and sound “just‐right” (strength = 2.73) exhibited the highest centrality, while other SP subtypes showed more modest or selective connectivity. Anxiety was the primary bridge symptom (bridge strength = 1.60), with the strongest cross‐domain link between “look ‘just‐right’” and “ordering.” The network architecture remained stable across subgroups, though elevated SP severity significantly increased global connectivity (*S* = 5.343, *p* = 0.002) without altering topology.

**Conclusion:**

SP may represent a clinically meaningful dimension of OCD psychopathology, within which SP subtypes operate as a heterogeneous set of experiences rather than a uniform construct, differentially shaping symptom connectivity. Anxiety serves as a critical bridge between SP and OC symptoms, while SP severity functions as a symptom network intensifier rather than a structural reorganizer. Future studies should explore whether precision interventions targeting anxiety and sensory‐channel‐specific pathways may improve treatment outcomes for refractory OCD with SP.

**Trial Registration:** Chinese Clinical Trial Registry: ChiCTR2100051536

## 1. Introduction

Obsessive‐compulsive (OC) disorder (OCD) is a diverse mental disorder, with a lifetime prevalence of around 2.4% in China [1]. Between 60% and 80% of patients report experiencing sensory phenomena (SP) [2, 3], characterized by distressing physical sensations, such as tingling or pressure, or mental sensations, such as feelings of incompleteness (i.e., internal tension/discomfort), preceding compulsions [2, 4]. Critically, SP‐positive OCD demonstrates distinct neurodevelopmental features, including an earlier age of onset, greater distress, and preferential association with symmetry/ordering symptoms [5–9], suggesting potential pathophysiological divergence from cognitive‐driven subtypes.

Prevailing cognitive‐behavioral models posit dysfunctional appraisals of intrusive thoughts as OCD’s core driver, with compulsions serving to prevent perceived harm [10–12]. However, these models may not fully account for repetitive behaviors driven by subjective sensory discomfort triggered by SP [2, 13, 14], as evidence indicates that the associations between obsessions and compulsions may differ among patients with and without SP. SP operate analogously to overt obsessions by triggering compulsions until sensory fulfillment is attained [13, 15]. Moreover, patients with SP often have difficulty identifying the obsessional thoughts associated with compulsive behaviors [16]. This phenomenological feature—the relative absence of identifiable obsessional triggers—may present a challenge for traditional CBT approaches, although exposure‐based interventions do not always require the explicit identification of obsession–compulsion linkages [17]. Taken together, these findings suggest that SP may be associated with disruptions in the traditional relationships between the core features of OCD [18]. However, no studies to date have directly examined how SP may impact the functional relationships between obsessions and compulsions.

To date, only a handful of studies have considered the influence of SP on symptom interrelationships in OCD. While meta‐analyses indicate SP relevance across all symptom dimensions [13], individual studies have found that SP predicts specific symptom dimensions more than others. For example, SP demonstrates stronger associations with symmetry/ordering symptoms [8, 19], whereas connections to contamination/washing symptoms remain inconsistent relative to harm‐avoidance motivations [19, 20].

Advancing our understanding of SP and OC symptom interrelationships requires analytical methods capable of mapping complex associations within symptom networks. In contrast to the traditional view of mental disorders as underlying entities that cause symptoms, the network approach emphasizes associations between the symptoms themselves [21]. This approach enables granular examination of individual symptom associations while statistically controlling for network‐wide interactions [22]. Critically, it identifies core symptoms that maintain network stability and bridging symptoms linking distinct symptom clusters [23]. Interventions targeting core and bridging symptoms within the symptom network can alter the structure of the psychiatric disorder network, thereby hindering the progression of psychiatric disorders [21, 23]. In comparison to research on other affective disorders, there are only a limited number of studies that have employed network analysis in the context of OCD. Furthermore, the existing studies primarily concentrate on the relationships between OCD and its comorbidities while largely overlooking SP [24–26]. The application of network analysis to identify core and bridging symptoms in OCD patients with SP is essential for understanding symptom heterogeneity, characterizing the impact of SP on these symptoms, identifying intervention targets, and improving intervention outcomes.

This study pioneers an investigation into the symptom network architecture of OCD patients with SP, postulating that SP represents a dimensional feature with potential neurodevelopmental underpinnings. Critically, we examine whether different SP subtypes show distinct patterns of association with specific OCD symptom dimensions. We aim to (a) identify the central and the bridge symptoms and (b) examine the difference in the symptom network across early/late‐onset and high/low SP‐severity subgroups. We hypothesize that SP symptoms will emerge as the network’s core hub, activating obsessive thoughts predominantly via heightened anxiety as a mediating bridge and driving compulsive behaviors through modality‐specific sensory channels (e.g., visual/tactile), with emotion potentially serving as a critical bridge between sensory triggers and compulsions. Furthermore, we postulate that early‐onset OCD will demonstrate heightened SP‐compulsion integration due to neurodevelopmental underpinnings, while greater SP severity will proportionally modulate symptom interdependencies. With this research, we hope to provide novel perspectives that may ultimately inform intervention development and targeted neuromodulation protocols for OCD with SP.

## 2. Methods

### 2.1. Participants and Procedure

This cross‐sectional multicenter study enrolled 1758 OCD patients from April 2021 to March 2025 across 15 participating hospitals in China through the China OCD Cohort (COCC), with data collected via a specialized disease electronic platform. The inclusion criteria are as follows: (1) receive a formal diagnosis of OCD based on DSM‐5 criteria, confirmed by a senior psychiatrist with the rank of associate chief physician or above; (2) YBOCS total score of 16 or higher (or 10 or higher in single‐dimension cases); (3) age range of 18–60 years; and (4) provide written consent after being fully informed about the study. The selection process ruled out participants who (1) submitted incomplete or improperly documented assessment forms, potentially indicating they hadn’t fully grasped the survey questions, (2) duplicate questionnaire records, and (3) suffered from serious psychiatric conditions including bipolar mania, schizophrenia, intense suicidal thoughts or actions, or self‐harming behaviors (as confirmed by Mini‐International Neuropsychiatric Interview [M.I.N.I.] diagnosis), and (4) exhibited severe emotional distress (Beck Depression Inventory‐II [BDI‐II] scores of 29 or higher and/or Beck Anxiety Inventory [BAI] scores of 36 and above). After implementing these screening measures, our final analytical cohort comprised 1350 OCD patients.

Collected data encompassed demographic characteristics, clinical history, and assessment outcomes from standardized scales, including the OC Inventory‐Revised (OCI‐R), BAI, BDI‐II, Yale‐Brown Obsessive Compulsive Scale (Y‐BOCS), and University of São Paulo SP Scale (USP‐SPS). The study received approval from the Ethics Committee of the Nanjing Medical University Affiliated Brain Hospital (IRB Number 2021‐KY044‐01/2021–KY044‐01‐ND2024) and was registered with the International Clinical Trials Registry Platform. Each participant signed a written consent form after being given a comprehensive rundown of the study purpose and procedures. All personal identifiers were removed to ensure data confidentiality during the analysis.

### 2.2. Measures

#### 2.2.1. The USP‐SPS

The USP‐SPS is a semi‐structured scale designed to evaluate the presence and severity of various types of SP that manifest before or during the execution of repetitive behaviors. The scale has undergone validation in both Portuguese [27] and English versions [28] and consists of two components: a symptom checklist (binary items) and a severity scale (Likert‐type items). The symptom checklist comprises nine subtypes of SP examples, encompassing all previously documented SP types. Following the review of each example, participants indicated their experience with SP by responding “yes,” “no,” or “in the past” and provided additional documentation regarding the age of onset and any SP not included in the provided examples. The Chinese version was developed through a standardized forward‐backward translation procedure with expert panel review and pilot testing, conducted by our research team with authorization from the original scale developers (the Rosario team); its psychometric properties have been established in a prior validation study [3]. Given that the network analysis in the present study used only the binary checklist component, we assessed its internal consistency using the Kuder‐Richardson formula 20 (KR‐20). In the current multicenter OCD sample (*n* = 1350), the checklist (excluding item E) demonstrated good internal consistency (KR‐20 = 0.81), which is consistent with our prior validation study (KR‐20 = 0.80) [3]. This study focused on the interview results regarding the occurrence of a SP in the past month as the subject of the research. In previous validation, item E exhibited cross‐loading and conceptual incongruence with other factor items, leading to its exclusion from the current network analysis, which includes eight validated symptom subtypes.

#### 2.2.2. OCI‐R

Each dimension consists of three items evaluated using a 5‐point Likert scale, from 0 (not at all) to 4 (extremely), resulting in a total score range of 0–72 [29]. The validated Chinese version of the OCI‐R exhibits satisfactory psychometric properties [30].

#### 2.2.3. BAI [31]

The BAI serves as a self‐report instrument comprising 21 items designed to assess the intensity of anxiety symptoms encountered during the previous 7‐day period. Participants rate each item on a 4‐point scale, where 0 indicates “none” and 3 represents “severe,” which yields total scores spanning from 0 to 63. Notably, elevated scores correspond to greater anxiety symptom severity. Specifically, scores between 0 and 21 suggest mild anxiety levels, while those falling between 22 and 35 indicate moderate anxiety. A score of 36 or above; however, points to severe anxiety manifestations. The Chinese adaptation of this scale has undergone rigorous validation and demonstrates robust psychometric characteristics [31, 32].

#### 2.2.4. BDI‐II [33]

The BDI‐II evaluates depressive manifestations across 21 items, employing a 0–3 point scale for each response, which ultimately yields a total score between 0 and 63. Individuals scoring 0–13 fall within the normal range, whereas scores of 14–19 suggest mild depressive symptoms, 20–28 indicate moderate depression, and 29–63 point to severe depressive states. The Chinese adaptation of this assessment tool has proven its worth as both a reliable and valid instrument for gauging the intensity of depression [33].

#### 2.2.5. M.I.N.I. [34, 35]

The M.I.N.I. is a brief, structured diagnostic interview codeveloped by psychiatrists and clinicians in the United States and Europe for the assessment of DSM‐IV and ICD‐10 psychiatric disorders. Intended for multicenter clinical trials, epidemiological studies, and preliminary diagnostic evaluations in non‐research clinical environments, the administration duration was roughly 15 min. The validated Chinese version exhibits strong psychometric properties.

### 2.3. Statistical Analysis

Utilizing the USP‐SPS, patients with OCD were categorized into two groups through semi‐structured interviews: those exhibiting SP (SP‐positive) and those not exhibiting them (SP‐negative). Statistical analyses were conducted utilizing R (version 4.5.0). Data missingness (< 5%) was addressed through imputation via chained equations (utilizing the mice package in R), followed by recalculation of derived variables after the imputation process. Independent *t*‐tests were utilized for group comparisons of continuous variables, applying Welch’s *t*‐test in cases of heteroscedasticity. For categorical gender distributions, *χ* ^2^ tests were conducted, alongside the calculation of Cohen’s *d* and φ effect sizes with 95% confidence intervals (CIs) (*α* = 0.05). The network analysis adhered to the four‐step framework proposed by Zhao et al. [36], which includes (1) network estimation, (2) calculation of centrality indices, (3) assessment of stability, and (4) comparison of networks.

#### 2.3.1. Estimation of Networks

The mixed graphical model (MGM), introduced by van Borkulo et al. [37], was employed to estimate the network structure. This estimation method was suitable for both nonbinary (OC/anxiety‐depressive symptoms) and binary (SP) data. The R package “MGM” implemented LASSO regularization via penalized maximum likelihood estimation [38, 39], with hyperparameter selection through 10‐fold cross‐validation (lambdaSel = “CV", lambdaFolds = 10). AND‐rule edge selection shrunk small correlations to zero, generating sparse networks [40–42]. Node predictability was quantified as *R* ^2^ for continuous variables and marginal correct classification (CCmarg) for binary variables [43]. Networks were visualized in Qgraph using a spring layout with symptom domains grouped (USP‐SPS, OCI‐R, BAI, and BDI) [44]. Edges were colored by association direction (red = positive and blue = negative), node borders were weighted by prediction accuracy, and layout consistency was maintained through the AverageLayout function [45]. As a sensitivity check, we also estimated the network using EBIC (*γ* = 0.25); the results were highly consistent (Table S5).

#### 2.3.2. Centrality Estimation

Centrality indices for a node within the network were calculated using the “centralityPlot” function from the R package “qgraph.” Four centrality metrics are frequently employed: strength, expected influence, betweenness, and closeness. Strength and expected influence were associated with directed connectivity, whereas betweenness and closeness were indicative of indirect connectivity [46]. The strength is defined as the total of the absolute edge weights associated with a node [47]. While strength is typically viewed as a dependable centrality metric in networks devoid of negative edges, in networks exhibiting negative correlations, expected influence—which incorporates the sign of edge weights—may offer a more precise assessment of a node’s centrality [46, 47]. Furthermore, our network comprised three symptom communities—SP, OC symptoms, and anxiety‐depressive symptoms; therefore, bridge strength was calculated to assess the connectivity of symptoms between different communities [48]. The activation of symptoms associated with increased bridge strength may facilitate comorbidity among the symptom domains.

#### 2.3.3. Accuracy and Stability Estimation

Given the sampling variability inherent in the estimated network model, assessing the accuracy and stability of edge weights and centrality indices is crucial [49]. We used the R package “bootnet” to perform bootstrap analyses [42, 49]. By resampling with replacement, 95% CIs for parameter estimates were constructed to evaluate edge‐weight accuracy, with narrower CIs indicating greater reliability [50]. However, since centrality indices rely on absolute edge weights, their stability cannot be assessed solely by CIs [42]. Therefore, the case‐drop bootstrap was applied to test centrality stability by correlating indices from the whole and subset samples. Centrality metrics were considered stable if correlations remained high after randomly excluding up to 70% of the data. Stability was quantified using correlation stability (CS) coefficients, with recommended thresholds of 0.5 or higher and a minimum of 0.25 [42]. Additionally, bootstrapped difference tests assessed whether differences between edges or centrality indices were significant by checking if their 95% CIs included zero. All bootstrapping procedures were conducted 2000 times.

#### 2.3.4. Network Comparison

The network comparison test (NCT) from the R package “NetworkComparisonTest” was applied to assess the differences between the two estimated network models using a permutation approach [51]. Differences in global strength and edge weights were evaluated to capture variations in the overall and local network properties. Global strength, defined as the sum of all edge weights [46], reflects symptom connectivity and disorder vulnerability, with higher values indicating stronger inter‐symptom associations [52]. Local differences pertain to variations in individual edge weights or centrality measures. Notably, this study conducted comparative network analysis across (1) early‐onset (≤17 years) versus late‐onset (>17 years) subgroups and (2) high‐SP versus low‐SP subgroups based on the median split.

## 3. Results

### 3.1. Descriptive Analysis of Symptoms

Independent sample *t*‐tests and Pearson’s chi‐square tests were performed to compare demographic and clinical characteristics between SP‐positive (*n* = 850) and SP‐negative (*n* = 500) individuals. The SP‐positive group exhibited a significantly younger age compared to the negative group (27.6 years [SD = 8.13] vs. mean age 29.7 years [SD = 10.2]; *d* = 0.23, *p* < 0.001) and reported an earlier age of symptom onset (20.3 years [SD = 7.85] vs. 22.4 years [SD = 9.76]; *d* = 0.25, *p* < 0.001). No significant differences were observed in sex distribution or illness duration.

Clinically, the SP‐positive group demonstrated significantly higher OC symptom severity on the OCIR scale (*d* = −0.40, *p* < 0.001), with notable differences across specific dimensions: washing, hoarding, ordering, checking, and neutralizing (all *d* = −0.23 to −0.45, *p* < 0.001). However, obsessing symptoms did not differ significantly between groups (*d* = −0.10, *p* = 0.082).

Although total YBOCS scores were comparable (*d* = −0.10, *p* = 0.079), the SP‐positive group exhibited greater compulsion severity (*d* = −0.16, *p* = 0.010). For comorbid conditions, depression severity (BDI) was higher in the SP‐positive group (*d* = −0.16, *p* = 0.006), whereas anxiety symptoms (BAI) showed no significant difference (*d* = −0.07, *p* = 0.192). Complete descriptive statistics are presented in Table 1.

**Table 1:** Comparative analysis of demographic and clinical characteristics in OCD patients with and without sensory phenomena (SP).

Variable	SP‐negative (*N* = 500)	SP‐positive (*N* = 850)	Group difference	*p*
	Mean (SD)/*n* (%)	Mean ± SD/*n* (%)	Effect size (95% CI)	—
Demographics				
Male^b^	288 (57.6%)	461 (54.2%)	*φ* = 0.04 (−0.01, 0.09)	0.144
Female^b^	205 (41.0%)	387 (45.5%)		
Age^a^	29.7 (10.2)	27.6 (8.13)	0.23 (0.12, 0.34)	<0.001 ^∗∗∗^
Age of onset^a^	22.4 (9.76)	20.3 (7.85)	0.25 (0.14, 0.36)	<0.001 ^∗∗∗^
Course	7.36 (7.50)	7.46 (6.83)	−0.01(−0.12, 0.10)	0.860
OCIR	19.9 (11.0)	25.2 (12.8)	−0.40 (−0.51, −0.29)	<0.001 ^∗∗∗^
Washing	3.72 (3.55)	4.93 (3.79)	−0.30 (−0.41, −0.19)	<0.001 ^∗∗∗^
Obsessing	5.46 (2.95)	5.78 (3.09)	−0.10 (−0.209, 0.012)	0.082
Hoarding^a^	1.97 (2.17)	2.63 (2.59)	−0.25 (−0.36, −0.14)	<0.001 ^∗∗^
Ordering^a^	2.44 (2.36)	3.79 (2.91)	−0.45 (−0.56, −0.34)	<0.001 ^∗∗∗^
Checking^a^	3.85 (3.02)	4.65 (3.26)	−0.23 (−0.34, −0.12)	<0.001 ^∗∗∗^
Neutralizing^a^	2.42 (2.64)	3.49 (2.95)	−0.34 (−0.46, −0.23)	<0.001 ^∗∗∗^
YBOCS	21.3 (7.29)	22.0 (6.94)	−0.10 (−0.209, 0.012)	079
Obsessions	11.2 (3.86)	11.3 (3.64)	−0.01 (−0.122, 0.099)	0.833
Compulsions	10.1 (4.76)	10.7 (4.11)	−0.16 (−0.269, −0.048)	0.010 ^∗^
BAI	14.6 (10.1)	15.4 (10.5)	−0.07 (−0.184, 0.037)	0.192
BDI	16.3 (11.2)	18.1 (12.0)	−0.16 (−0.266, −0.045)	0.006 ^∗∗^

^a^Welch’s *t*‐test.

^b^Pearson’s chi‐square test.

^∗^ *p* < 0.05.

^∗∗^ *p* < 0.01.

^∗∗∗^ *p*  < 0.001.

### 3.2. Symptom Network of OC and SP

#### 3.2.1. Estimation of Networks

**Figure 1:**
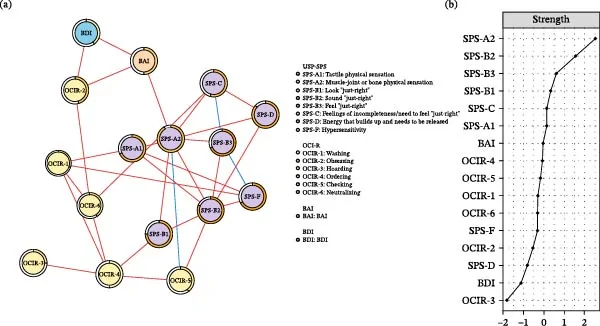
Symptom network and centrality of SP‐positive OCD (*N* = 850). (a) Network structure. Nodes represent symptoms: USP‐SPS (purple), OCI‐R (yellow), BAI (orange), and BDI (blue). Edges indicate partial correlations among symptoms, with the thickness of the edges reflecting the strength of the associations (Red, positive; blue, negative). Edges are displayed only if their weight exceeds 0.15. (b) Symptom strength ranking.

Figure 1 illustrates the symptom network and strength centrality. Out of 120 edges, 58 were eliminated. The sparsity of the network was 0.52. The most significant associations were identified between “tactile physical sensation” and “muscle‐joint or bone physical sensation,” as well as between “look ‘just‐right’” and “sound ‘just‐right’,” indicating the strongest within‐domain correlations. The primary cross‐dimensional connectivity associated with SP and OC symptoms revealed that “look ‘just‐right’” exhibited the most significant correlation with “ordering”. The edge weights are given in Table S1. Node predictability metrics are reported in Table S4. Sensitivity analyses confirmed that findings were robust to the choice of regularization strategy, with edge weights and centrality rankings highly consistent between CV‐ and EBIC‐estimated networks (*r* = 0.96 and Spearman’s ρ = 0.90, respectively; Table S5).

#### 3.2.2. Centrality Estimations

**Figure 2:**
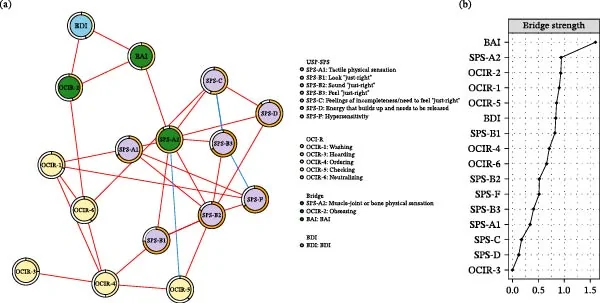
Bridge symptom network and bridge strength centrality in SP‐positive OCD (*N* = 850). (a) Network structure. Nodes represent symptoms: USP‐SPS (purple), OCI‐R (yellow), BDI (blue), and Bridge Symptoms (green). Edges represent partial correlations between symptoms, with edge thickness proportional to the strength of the association (Red, positive; blue, negative). Only edges with |weight| > 0.15 are shown. (b) Bridge strength centrality ranking.

Figure 2 displays the ranked distributions of bridge strength centrality for OC and SP symptoms. SP symptoms dominated strength centrality rankings, with “muscle‐joint or bone physical sensation” (strength = 3.41), “sound ‘just‐right’” (strength = 2.73), and “feel ‘just‐right’” (strength = 2.05) demonstrating the highest values. Centrality difference tests confirmed these symptoms exhibited significantly greater strength than others (*p*  < 0.05), while “energy that builds up and needs to be released,” “depression,” and “hoarding” showed the lowest strength values. Among statistically significant cross‐symptom cluster connections identified by edge weight difference tests (*p*  < 0.05), the three strongest are: (1) “look ‘just‐right’” and “ordering,” (2) “muscle‐joint or bone physical sensation” and BAI, and (3) “tactile physical sensation” and “washing.” (statistical details are shown in Figure S1).

The centrality strength highlights the importance of nodes within the comorbidity network, indicating that interventions aimed at these central symptoms may mitigate the overall severity of comorbidity. Bridge centrality pertains to symptoms that connect various disorders, and metrics of bridge symptoms are useful for evaluating the bridge effect. Among bridge symptoms, “anxiety” (bridge strength = 1.60), “muscle‐joint or bone physical sensation” (bridge strength = 0.95), and “obsessing” (bridge strength = 0.93) emerged as the most prominent. Tests of bridge strength differences (Figure S1) indicated significant variations in nearly half of the symptoms, identifying “anxiety” as the principal bridge node connecting SP and OC symptoms. Comprehensive centrality metrics can be found in Table S2 (strength) and Table S3 (bridge strength).

#### 3.2.3. Accuracy and Stability Estimation

This study prioritizes the stability and accuracy of the network structure. Figure S2 presents the estimated CIs for edge weights derived from bootstrapping methods. The narrow CIs suggest a satisfactory level of accuracy. Figure S3 presents the outcomes of the centrality‐stability estimation procedure. The CS coefficients for both strength and bridge strength centrality surpassed 0.5, demonstrating their reliability in assessing the characteristics of network nodes. The CS coefficients for closeness, betweenness, bridge closeness, and bridge betweenness centrality were 0.594, 0.516, 0.361, and 0.594, respectively, as illustrated in Figure S4.

#### 3.2.4. Network Comparison

**Figure 3:**
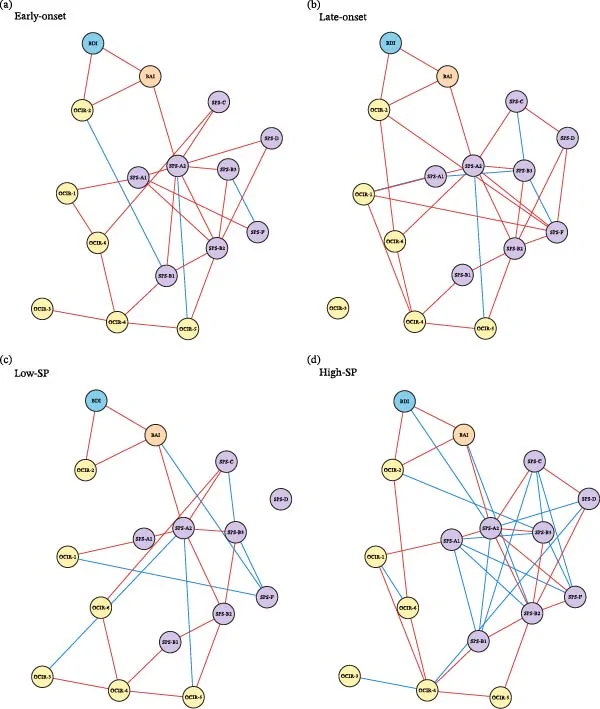
Symptom network comparisons. (a) Early‐onset (≤17y, *n* = 342) versus (b) late‐onset (>17y, *n* = 508); (c) high‐SP (*n* = 417) versus (d) low‐SP (*n* = 433). Edges represent partial correlations between symptoms, with edge thickness proportional to the strength of the association (Red, positive; blue, negative). Only edges with |weight| > 0.15 are shown.

We compared network structures between early‐onset and late‐onset patient groups (Figure 3), finding no significant differences in overall network structure (*M* = 0.544, *p* = 0.493) or global strength (early‐onset: 11.270, late‐onset: 13.488; *S* = 2.218, *p* = 0.260). Complementary analyses comparing high‐SP and low‐SP groups based on median split (Figure 3) demonstrated similarly preserved network topology (*M* = 0.459, *p* = 0.759), but identified significantly stronger global connectivity in the high‐SP group (high‐SP: 14.665, low‐SP: 9.322; *S* = 5.343, *p* = 0.002). Crucially, both group comparisons consistently indicated nonsignificant differences in specific edge weights (all *p*  > 0.05 after FDR correction) and in centrality metrics across strength, closeness, betweenness, and bridge strength (all *p*  > 0.05).

## 4. Discussion

This study conducted network analysis for the first time to elucidate relationships between SP and OC symptoms in a multicenter clinical cohort of 1350 OCD patients, among whom SP‐positive cases constituted 63.0% (*n* = 850). Crucially, SP symptoms—particularly “muscle‐joint or bone physical sensation” and “sound ‘just‐right’"—demonstrated significantly greater strength centrality than core OC symptoms. Notably; however, SPs did not operate as a uniform construct: different SP subtypes showed distinct centrality profiles and specific patterns of association with OCD symptom dimensions, underscoring their heterogeneity rather than a monolithic influence. Furthermore, elucidating the connection between SP and OC dimensions, anxiety (BAI) emerged as the primary bridge symptom linking SP and OC dimensions, while the strongest cross‐domain connection was identified between perceptual ("look ‘just‐right’") and compulsive ("ordering") symptoms. Critically, the core network architecture remained stable across early‐ and late‐onset groups and across high‐ and low‐SP severity subgroups, with elevated SP severity amplifying global connectivity without altering the network topology. These findings collectively underscore the fundamental role of SP as a clinically meaningful dimension of OCD psychopathology.

This study identified, through between‐group comparisons, that OCD patients with SP exhibit a distinct clinical phenotype characterized by a significantly earlier age of onset, more severe compulsive behaviors, and higher levels of depression. These differences likely reflect SP as a dimensional feature that varies in severity across patients. Network analysis further confirmed the central role of SP symptoms within the overall symptom structure: “muscle‐joint or bone physical sensation” (strength = 3.41), “sound ‘just‐right’” (strength = 2.73), and “feel ‘just‐right’” (strength = 2.05) demonstrated significantly higher centrality than traditional OC symptoms (OCI‐R strengths 0.36–1.58). This finding aligns with Stamatis [18] (2021, Study 2), where SP symptoms occupied highly central positions within the OCD pathological network. Notably, Stamatis (Study 1) identified four distinct subgroups among high‐SP patients using latent class analysis (LCA), with Class 3 and Class 4 both exhibiting strong associations with symmetry symptoms, suggesting that SP subtypes may drive specific OCD dimensions through distinct sensory channels [2, 53, 54]. The high centrality of “muscle‐joint/bone physical sensation” and “sound ‘just‐right’” indicates interoceptive processing dysfunction, implying compromised thalamocortical processing, potentially attributable to functional abnormalities in the basal ganglia–thalamic sensorimotor loop and the superior temporal gyrus–prefrontal auditory regulation circuit [55–57]. Alternatively, SP may serve as vulnerability markers, potentiating obsessive cognition in response to cognitive triggers. It is important to note; however, that not all SP subtypes contributed equally to this centrality pattern—“muscle‐joint/bone physical sensation” and “sound just‐right” were particularly prominent, while other SP subtypes showed more modest or selective connectivity, consistent with the view that SPs represent a heterogeneous set of phenomenological experiences. Collectively, these findings suggest that, in the SP‐positive subtype, SP may complement—rather than replace—cognitive processes as pathological drivers. While obsessive thoughts remain important in many patients, SP appears to constitute an additional driving mechanism, necessitating concurrent clinical assessment and intervention for both SP and obsessive cognition. This driving role may originate from aberrant sensory regulation during development: Tonna et al. [58] found that SP manifests as a “trait‐like state” in childhood, directly linked to developmental dysfunction within the basal ganglia–thalamic sensorimotor loop [56]. Their research posits compulsive behaviors as a “compensatory mechanism for sensory imbalance,” providing a neurodevelopmental explanatory framework for the centrality of SP observed in this study.

Previous studies indicate a higher severity of hoarding symptoms in OCD patients with SP [2, 18, 59], consistent with our between‐group comparison results. However, within the network structure, hoarding symptoms exhibited a peripheral profile (strength = 0.36). The weak connectivity between hoarding and the core SP‐OC symptom pathways suggests that it may constitute a heterogeneous dimension independent of the core SP‐OC pathology. It is important to note that hoarding can manifest not only as an OCD symptom but also as an independent disease entity (e.g., hoarding disorder) or as a symptom of other conditions (e.g., dementia, neurodevelopmental disorders, and schizophrenia) [24, 60–62]. Therefore, the low centrality of hoarding in this study likely reflects its distinct pathological mechanism rather than lower symptom severity.

A key contribution of the present network analysis is the demonstration that SP–OCD coupling is selective rather than generalized. Network analysis revealed, for the first time at the symptom level, mechanisms of coupling between SP and OC symptoms via specific sensory‐behavioral pathways. The strongest cross‐dimensional connections were “look ‘just‐right’→ordering” (highest edge weight) and “tactile physical sensation→washing,” consistent with Stamatis [18] (2021, Study 2) and clinical observation. This indicates that different SP subtypes may drive specific compulsive behaviors through distinct neural circuits. Specifically, the connection between visual SP and symmetry symptoms may reflect environmental disorganization stemming from occipito‐parietal spatial processing deficits, which subsequently trigger compensatory ordering behaviors—a mechanism distinct from traditional harm‐avoidance models. Vellozo et al. [63] confirmed in a large sample that the symmetry dimension is present in 86.8% of OCD patients and is the earliest‐onset symptom subtype. The connection between tactile SP and washing behavior demonstrates complexity: Stamatis et al. observed that washing behavior was significantly correlated not only with tactile discomfort but also with visual “just‐right” experiences. While prior research primarily focused on differences between SP and harm‐avoidance motivation (e.g., washing can be driven by either mechanism) [20, 21, 60, 62, 63], Stamatis [18] further noted heterogeneity within SP‐driven washing subgroups. In contrast, our network did not reveal significant connections between the washing behavior and other SP symptoms or anxiety. This discrepancy may arise from the specificity of our SP‐positive sample: after excluding SP‐negative patients, the driving effect of tactile SP on washing became more pronounced, potentially linked to sensory gating deficits [18, 64, 65] and insular‐prefrontal sensory integration abnormalities [66]. Together, these pathway differences suggest the hypothesis that SP may function as a precursor factor in OCD. If confirmed in longitudinal studies, exposure therapy targeting specific sensory channels (e.g., visual/tactile) could potentially block symptom propagation more precisely, though this remains to be tested in treatment research.

Although SP patients exhibited greater depression severity (BDI *d* = −0.16, *p* = 0.006), network analysis identified anxiety symptoms (BAI) as a key bridge node with a significantly higher bridge strength value (strength = 1.60) than other nodes (*p*  < 0.05). This phenomenon may be closely related to the core motivational symptom of harm avoidance, with prior studies confirming a specific association between harm avoidance and anxiety [67, 68], which suggests that trait anxiety may enhance symptom network connectivity in the clinical OCD sample via the harm avoidance mechanism. Potential pathways include: (1) when obsessive thought‐behavior connectivity weakens in high‐SP patients, anxiety might accelerate the transformation of SP into compulsive behaviors by amplifying threat appraisal of somatic sensations via the amygdala–anterior insula; (2) decision‐making impairments in SP patients, as revealed by Stamatis [18] (Study 3), involving higher cognitive circuit (e.g., anterior cingulate cortex) dysfunction, may concurrently intensify anxiety and the efficiency of sensory‐behavior transformation [54]. Furthermore, depressive symptoms occupied a peripheral node position within the network, showing significant connections only with anxiety and obsessions. This pattern aligns with the secondary nature of OCD–MDD comorbidity: Belli et al. [69]’s large‐scale study found OCD preceded MDD in 65.7% of cases. Depressive symptoms in OCD primarily arise via nonspecific functional impairments (e.g., “withdrawal from daily activities and deprivation of social rewards” [70, 71])and anxiety‐mediated cognitive appraisals (e.g., “negative interpretations of intrusive thoughts” [72, 73]. Although obsessive beliefs correlate with depression, their predictive power for depression significantly diminishes after controlling for anxiety/avoidance factors [73], providing a mechanistic explanation for the peripheral network position of depression.

Despite the early‐onset group (age of onset ≤17 years) being younger and having a more prolonged illness duration, network comparison tests revealed no significant differences in network structure invariance (*M* = 0.544, *p* = 0.493), global strength (*S* = 2.218, *p* = 0.260), or any node strength/edge weights. Analysis of high‐versus low‐SP subgroups similarly demonstrated preserved network topology (*M* = 0.459, *p* = 0.759), reinforcing structural stability as a core characteristic of SP–OCD. However, the high‐SP group exhibited significantly stronger global connectivity (*S* = 5.343, *p* = 0.002), suggesting that heightened SP severity intensifies overall symptom interactions. Notably, while no edge weights or centrality metrics showed significant between‐group differences after FDR correction (all *p*  > 0.05), the high‐SP group exhibited a marginally significant trend toward stronger connectivity, specifically in the SPS‐B2‐BAI edge (*E* = 0.248, *p* = 0.066, uncorrected). This pattern indicates that the core symptom interaction architecture in SP–OCD remains stable across different developmental stages and SP severity. While this stability is consistent with the possibility of a neurodevelopmental origin, cross‐sectional data alone cannot establish a developmental etiology; longitudinal studies are needed to address this question. At the same time, elevated SP severity intensifies global network connectivity without restructuring its core architecture, supporting SP’s role as an “intensifier” rather than a “structural reorganizer” of the symptom network. These findings align with Tonna et al.’s [58] hypothesis that compulsive behaviors represent a “pathological persistence of adaptive responses from early development,” where sensory regulatory deficits not only perpetuate behavioral patterns into adulthood but also amplify system‐wide symptom coupling. Future longitudinal studies are needed to validate the temporal stability of these core pathways.

## 5. Limitations

Several limitations should be noted. First, although severe psychiatric comorbidities were excluded, the inclusion of participants with moderate anxiety and depression may have influenced the network structure. Second, the binary classification of SP (present/absent) overlooks dimensional severity gradients; the exclusion of item E due to cross‐loading further limits the measurement precision. Third, the high/low‐SP subgroup comparison relied on median‐split grouping rather than data‐driven clustering, potentially obscuring more nuanced phenotypic stratifications. Fourth, the cross‐sectional design precludes causal inference; longitudinal studies are needed to delineate SP–symptom dynamics. Fifth, the centrality of SP nodes may be partially inflated by two factors: (a) the restriction of the analysis to SP‐positive patients and (b) greater within‐domain clustering among the eight conceptually related SP subtypes relative to the more heterogeneous OCD dimensions. An exploratory network analysis in SP‐negative patients (*n* = 500; Figure S5) was conducted as a qualitative reference, though a direct statistical comparison was precluded by differing node compositions. Future studies incorporating SP‐negative patients and employing more balanced node compositions would help clarify the generalizability of these centrality findings. Finally, while network analysis offers novel insights into SP–OCD heterogeneity, its application here remains exploratory given ongoing methodological debates about stability and generalizability [74–76].

## 6. Conclusion

This study identifies SP as a central and distinct pathological feature in an OCD subpopulation while demonstrating that different SP subtypes operate as heterogeneous experiences with selective—rather than generalized—associations with specific OC symptom dimensions. These findings suggest that SP represents a clinically meaningful dimension of OCD psychopathology. Network analysis revealed, for the first time, symptom‐level coupling between SP and OC symptoms through specific sensory‐behavioral pathways, suggesting that SP may complement cognitive processes as an additional pathological driver in this subgroup. Notably, while comorbid depression severity was elevated in SP patients, anxiety demonstrated more potent bridge properties, indicating its potential role in linking sensory and cognitive domains. The core symptom architecture remained stable across different developmental stages and SP severity. This stability may reflect a neurodevelopmental origin of SP, though developmental measures and longitudinal studies are required to verify this hypothesis. In this context, SP amplifies global network connectivity without altering the topology, functioning as a symptom “intensifier” rather than a “structural reorganizer.”

These findings suggest the hypothesis that targeting SP symptoms (e.g., via sensory channel‐specific exposure therapies) and anxiety modulation could disrupt symptom pathways. Future research should explore neuromodulation addressing sensorimotor and anxiety‐related neural circuits and empirically test sensory‐focused interventions tailored to this OCD subtype, which may inform precision medicine approaches for treatment‐resistant cases.

## Author Contributions

The corresponding author (Na Liu, principal investigator) certifies the accuracy of contributions and confirms collective agreement with the journal policies. Conceptualization, data curation, validation, writing – review and editing: Na Liu, Yue Sun, and Xuedi Zhang. Formal analysis, methodology, software, visualization, writing – original draft: Yue Sun. Funding acquisition, investigation (coordinating investigator), project administration, supervision: Na Liu. Site investigators: Wenxin Tang, Qianqian Li, Jun Yan, Si Mi, Zhanjiang Li, Bin Li, Guiyun Xu, Congwen Yang, Maorong Hu, Zhenqing Zhang, Yanbin Jia, Zhen Tang, Xiaoping Wang, Jun Ma, Changhong Wang, and Wei Liu. Resources: Na Liu, Wenxin Tang, Qianqian Li, Jun Yan, Si Mi, Zhanjiang Li, Bin Li, Guiyun Xu, Congwen Yang, Maorong Hu, Zhenqing Zhang, Yanbin Jia, Zhen Tang, Xiaoping Wang, Jun Ma, Changhong Wang, and Wei Liu.

## Funding

This study was funded by the National Natural Science Foundation of China Youth Program (Grant 81901390), Jiangsu Provincial Key R&D Program‐Social Development Special Project (Grant BE2021616), and Jiangsu Provincial Social Development Project‐General Program (Grant BE2022678).

## Disclosure

All authors have made substantial contributions according to the CRediT taxonomy as detailed in the contributions table; have refined and approved the submitted version of the manuscript; and agree to be accountable for all aspects of the work.

## Conflicts of Interest

The authors declare no conflicts of interest.

## Supporting Information

Additional supporting information can be found online in the Supporting Information section.

## Supporting information


**Supporting Information** The supplementary material contains five figures and five tables supporting the main analyses. Figures S1–S4 present the bootstrap‐based robustness analyses of the primary symptom network (*N* = 850): edge weight and centrality difference tests (Figure S1), edge weight accuracy estimation (Figure S2), centrality index stability estimation via case‐drop bootstrapping (Figure S3), and plots of closeness, betweenness, bridge betweenness, and bridge closeness (Figure S4). Figure S5 shows the symptom network for SP‐negative OCD patients (*n* = 500) as a subgroup analysis. Tables S1–S4 report the detailed quantitative results, including complete edge weight values (Table S1), raw values of centrality and bridge centrality indices (Tables S2 and S3), and node predictability metrics (Table S4). Table S5 presents a sensitivity analysis comparing CV‐ and EBIC‐based network estimation.

## Data Availability

The data that support the findings of this study are available upon request from the corresponding author. The data are not publicly available due to privacy or ethical restrictions.
