# Supplementary material for: Network Analysis of Sensory Phenomena and Obsessive‐Compulsive Symptoms in OCD: A Multicenter Study
Source: Depress Anxiety. 2026 Aug 26;2026:1367751. doi: 10.1155/da/1367751 (PMC13507714; doi:10.1155/da/1367751)
Supplement: Supplementary file 1 [file DA-2026-1367751-s001.docx]

**Supplementary Material**

**Figure S1**. Difference test results for strength, bridge strength, and edges, using the non-parametric bootstrapping method (N = 850), where black grids indicate statistically significant differences and gray grids indicate non-significant differences. SPS-A1: Tactile Physical Sensation; SPS-A2: Muscle-joint or bone physical sensation; SPS-B1: Look "just-right"; SPS-B2: Sound "just-right"; SPS-B3: Feel "just-right"; SPS-C: Feelings of incompleteness/need to feel "just-right"; SPS-D: Energy that builds up and needs to be released; SPS-F: Hypersensitivity; OCIR-1: Washing; OCIR-2: Obsessing; OCIR-3: Hoarding; OCIR-4: Ordering; OCIR-5: Checking, OCIR-6: Neutralizing; BAI: Anxiety; BDI: Depression.

**Figure S2** Accuracy estimations of all edge weights using the non-parametric bootstrapping method (N = 850). Narrower confidence intervals (CIs) indicate higher reliability. The left panel displays the full accuracy test results, and the right panel shows edges with confidence intervals excluding zero.

**Figure S3** Stability estimations of centrality indices using the case-drop bootstrapping method (N = 850).

**Figure S4:** The plot of centrality indices closeness, betweenness, bridge betweenness, and bridge closeness of every symptom (N = 850).

**Figure S5.** Symptom network of SP-negative OCD patients (n = 500). Nodes represent the six OCI-R dimensions (yellow), anxiety/BAI (orange), and depression/BDI (blue). Edges represent partial correlations between symptoms (red = positive, blue = negative). Node size reflects strength centrality.

**Table S1** Values of edge weights between each of the OC symptoms, SP, and depression/anxiety.

Note: SPS-A1: Tactile Physical Sensation; SPS-A2: Muscle-joint or bone physical sensation; SPS-B1: Look "just-right"; SPS-B2: Sound "just-right"; SPS-B3: Feel "just-right"; SPS-C: Feelings of incompleteness/need to feel "just-right"; SPS-D: Energy that builds up and needs to be released; SPS-F: Hypersensitivity; OCIR-1: Washing; OCIR-2: Obsessing; OCIR-3: Hoarding; OCIR-4: Ordering; OCIR-5: Checking, OCIR-6: Neutralizing; BAI: Anxiety; BDI: Depression.

**Table S2:** The raw values of strength, closeness, and betweenness. (N = 850)

|  | strength | closeness | betweenness |
| --- | --- | --- | --- |
| SPS-A1 | 1.7380 | 0.0126 | 22 |
| SPS-A2 | 3.4138 | 0.0148 | 84 |
| SPS-B1 | 1.8626 | 0.0128 | 36 |
| SPS-B2 | 2.7268 | 0.0125 | 32 |
| SPS-B3 | 2.0547 | 0.0123 | 4 |
| SPS-C | 1.7382 | 0.0116 | 4 |
| SPS-D | 1.0720 | 0.0102 | 0 |
| SPS-F | 1.4139 | 0.0090 | 0 |
| OCIR-1 | 1.4281 | 0.0093 | 0 |
| OCIR-2 | 1.2614 | 0.0081 | 4 |
| OCIR-3 | 0.3623 | 0.0060 | 0 |
| OCIR-4 | 1.5873 | 0.0112 | 34 |
| OCIR-5 | 1.5230 | 0.0105 | 0 |
| OCIR-6 | 1.4200 | 0.0087 | 4 |
| BAI | 1.6034 | 0.0111 | 38 |
| BDI | 0.8424 | 0.0082 | 0 |

**Table S3:** The raw values of bridge strength, bridge betweenness, and bridge closeness. (N = 850)

|  | bridge strength | bridge closeness | bridge betweenness |
| --- | --- | --- | --- |
| SPS-A1 | 0.3421 | 8 | 0.1553 |
| SPS-A2 | 0.9463 | 34 | 0.1808 |
| SPS-B1 | 0.8219 | 18 | 0.1613 |
| SPS-B2 | 0.5256 | 11 | 0.1432 |
| SPS-B3 | 0.4081 | 0 | 0.1384 |
| SPS-C | 0.1793 | 2 | 0.1367 |
| SPS-D | 0.1255 | 0 | 0.1208 |
| SPS-F | 0.5124 | 0 | 0.1072 |
| OCIR-1 | 0.9065 | 0 | 0.1520 |
| OCIR-2 | 0.9321 | 2 | 0.1358 |
| OCIR-3 | 0.0000 | 0 | 0.0866 |
| OCIR-4 | 0.7120 | 13 | 0.1661 |
| OCIR-5 | 0.8528 | 0 | 0.1748 |
| OCIR-6 | 0.6603 | 0 | 0.1217 |
| BAI | 1.6034 | 17 | 0.1664 |
| BDI | 0.8424 | 0 | 0.1227 |

**Table S4** Network node predictability metrics. (N = 850)

| Variable | R2 | CC | nCC | CCmarg |
| --- | --- | --- | --- | --- |
| SPS-A1 | - | 0.731 | 0.129 | 0.691 |
| SPS-A2 | - | 0.922 | 0.132 | 0.910 |
| SPS-B1 | - | 0.793 | 0.204 | 0.740 |
| SPS-B2 | - | 0.847 | 0.198 | 0.809 |
| SPS-B3 | - | 0.689 | 0.004 | 0.690 |
| SPS-C | - | 0.626 | 0.187 | 0.540 |
| SPS-D | - | 0.887 | 0.000 | 0.887 |
| SPS-F | - | 0.701 | 0.090 | 0.671 |
| OCIR-1 | 0.294 | - | - | - |
| OCIR-2 | 0.451 | - | - | - |
| OCIR-3 | 0.245 | - | - | - |
| OCIR-4 | 0.495 | - | - | - |
| OCIR-5 | 0.374 | - | - | - |
| OCIR-6 | 0.403 | - | - | - |
| BAI | 0.495 | - | - | - |
| BDI | 0.438 | - | - | - |

Note: R² = proportion of variance explained (node predictability).

**Table S5** Sensitivity Analysis: Comparison of Network Estimation Using CV vs. EBIC Regularization.

| Metric | CV (10-fold) | EBIC (γ = 0.25) |
| --- | --- | --- |
| Sparsity | 0.52 | 0.65 |
| Edge weight correlation (r) | — | 0.964 |
| Strength centrality rank correlation (ρ) | — | 0.903 |

Note: CV = 10-fold cross-validation; EBIC = Extended Bayesian Information Criterion. Edge weight and centrality rank correlations are computed between CV- and EBIC-estimated networks.
